# Activation of VCAM-1 and Its Associated Molecule CD44 Leads to Increased Malignant Potential of Breast Cancer Cells

**DOI:** 10.3390/ijms15033560

**Published:** 2014-02-27

**Authors:** Pei-Chen Wang, Ching-Chieh Weng, You-Syuan Hou, Shu-Fang Jian, Kuan-Te Fang, Ming-Feng Hou, Kuang-Hung Cheng

**Affiliations:** 1 Institute of Biomedical Science, National Sun Yat-Sen University, Kaohsiung 80424, Taiwan; E-Mails: lovemessage1002@yahoo.com.tw (P.-C.W.); inoursky@gmail.com (C.-C.W.); poo779779@gmail.com (Y.-S.H.); chienfang1216@gmail.com (S.-F.J.); 2 Department of Research and Development, Eternal Chemical Co., Ltd., Kaohsiung 80778, Taiwan; E-Mail: CalvinFang@hotmail.com; 3 Department of Surgery, Kaohsiung Medical University Chung-Ho Memorial Hospital, Kaohsiung 80708, Taiwan; E-Mail: mifeho@kmu.edu.tw; 4 Department of Surgery, Kaohsiung Municipal Ta-Tung Hospital, Kaohsiung 80145, Taiwan; 5 Kaohsiung Medical University Joint Research Center, National Sun Yat-Sen University, Kaohsiung 80424, Taiwan

**Keywords:** *VCAM-1*, breast cancer progression, EMT, metastasis, chemoresistance

## Abstract

*VCAM-1* (CD106), a transmembrane glycoprotein, was first reported to play an important role in leukocyte adhesion, leukocyte transendothelial migration and cell activation by binding to integrin VLA-1 (α4β1). In the present study, we observed that *VCAM-1* expression can be induced in many breast cancer epithelial cells by cytokine stimulation *in vitro* and its up-regulation directly correlated with advanced clinical breast cancer stage. We found that *VCAM-1* over-expression in the NMuMG breast epithelial cells controls the epithelial and mesenchymal transition (EMT) program to increase cell motility rates and promote chemoresistance to doxorubicin and cisplatin *in vitro*. Conversely, in the established MDAMB231 metastatic breast cancer cell line, we confirmed that knockdown of endogenous *VCAM-1* expression reduced cell proliferation and inhibited TGFβ1 or IL-6 mediated cell migration, and increased chemosensitivity. Furthermore, we demonstrated that knockdown of endogenous *VCAM-1* expression in MDAMB231 cells reduced tumor formation in a SCID xenograft mouse model. Signaling studies showed that *VCAM-1* physically associates with CD44 and enhances CD44 and ABCG2 expression. Our findings uncover the possible mechanism of *VCAM-1* activation facilitating breast cancer progression, and suggest that targeting *VCAM-1* is an attractive strategy for therapeutic intervention.

## 1. Introduction

Breast cancer is the most common female malignancy in the world, accounting for more than 30% of all malignant tumors in women. Most research efforts on breast cancer have focused on familial predisposition to the disease, constituting 3%–10% of affected women [1,2]. Although understanding familial breast cancer provides an excellent model system for further studies, it is equally important to understand sporadic cases, which account for most breast cancer patients. Although the underlying molecular mechanisms of breast cancer pathogenesis remain mainly undiscovered, multiple genetic and epigenetic alterations have been connected to breast cancer, including the activation of oncogenes (MYC, ERBB2 and CCND1) [3–5] and the mutation or deletion of tumor suppressor genes (TP53 and CDH1) [6,7]. The advanced stage of human cancer is characterized by uncontrolled progression (malignancy) of the cancer from the site of origin to distant sites in a process called metastasis. Diagnosis of advanced stage of breast cancer is a devastating experience for both patient and family. Even so, research is lacking on the changes involved in the conversion of a benign tumor to an advanced stage of cancer.

The development and growth of cancer is a multi-stage process, and inflammation is the most important cause of cancer from initiation through progression [8,9]. The possible mechanisms by which inflammation can contribute to carcinogenesis include enhanced cell proliferation, alterations in epigenetic events and subsequent inappropriate gene expression, increasing resistance to apoptosis, and promotion of tumor neovascularization, invasion and metastasis [10,11]. Dramatic increase of pro-inflammatory mediators such as TNFα, IL-1, IL-6 and TGF-β cytokines, and chemokines combine with a distinct network of intracellular signaling molecules including upstream kinases and transcription factors to facilitate tumor promotion and progression [12–14]. When inflammation drives development of carcinogenesis, components of the tumor microenvironment, including epithelial tumor cells, stromal cells in the surrounding tissue, and infiltrated immune cells will release many inflammatory cytokines. These cytokines induce tumor cells to increase expression of some proinflammatory molecules such as P-selection, CXCR4, I-CAM1 and VCAM-1 adhesion molecules [15,16]. Many proinflammatory mediators, especially cytokines, chemokines and prostaglandins, turn on the malignant switches mainly controlled by vascular endothelial growth factor (VEGF) [17,18], thereby inducing inflammatory angiogenesis and invasion. Thus, proinflammatory mediators in carcinogenesis hold promise as potential targets for the chemoprevention of inflammation-associated carcinogenesis [19,20].

Vascular cell adhesion molecule-1 (VCAM-1), a 110-kDa transmembrane sialoglycoprotein and member of the immunoglobulin gene family, is mainly expressed on activated vascular endothelium [21,22]. *VCAM-1* is comprised of two isoforms in humans and mice which may have pathophysiologic implications. The full length form of *VCAM-1* contains seven Ig-like extracellular domains (7D VCAM-1) and is thought to be the predominant form expressed on the cell surface. Another isoform of VCAM-1 (6D VCAM-1) is an alternative splicing form lacking domain 4 [23]. *VCAM-1* is expressed constitutively or inducibly in many cell types, including some epithelia, mesothelium, endothelium, myoblast, dendritic cells and bone marrow stromal cells [24,25]. The secreted form of VCAM-1 occurs due to proteolytic cleavage released from the cell surface by the activity of neutrophil-derived serine proteases such as neutrophil elastase and cathepsin G or metalloproteases [26]. It has been reported that VCAM-1 is mainly involved in leukocyte transendothelial migration and leuokocyte retention into tissues [27,28]. For example, *VCAM-1* plays a central role in the recruitment of inflammatory cells, and its expression is rapidly induced by proinflammatory cytokines such as TNF-α, IL-6 and TGF-β1. VCAM-1 binds to integrin α4β1 on T lymphocytes [29,30]. Its soluble form has been reported to be chemotactic for T cells and monocytes, and angiogenic for endothelial cells [31,32]. In cultured human BEAS-2B bronchial epithelial cells, *VCAM-1* expression is induced by the cytokines interleukin-1, tumor necrosis factor and interleukin-4 [33]. *VCAM-1* expression on renal tubular epithelial cells has been demonstrated on biopsy sections recovered during acute renal allograft rejection [34]. Furthermore, *VCAM-1* over-expression in renal carcinoma is associated with tumor stage, tumor grade, overall survival and subtype of renal carcinoma (RCC) tumors [35,36]. Thus, *VCAM-1* expression may serve as a biomarker for patients with clear cell RCC. Together, these data suggest that VCAM-1 is a potential target for molecular intervention in carcinogenesis and requires further investigation.

In this study, we first observed the increasing expression of *VCAM-1* in breast cancer cells after inflammatory cytokine treatments. We further demonstrated that *VCAM-1* has a growth-promoting role in tumorigenesis *in vivo*, and furthermore promotes breast cancer migration and affects EMT mediated by TGF-β1 and IL-6. In addition, we also discovered that *VCAM-1* activation was involved in the development of chemoresistance in NMuMG breast cancer cells after exposure to low*-*dose doxorubicin *in vitro*, and *VCAM-1* may contribute to the activation of CD44 and ABCG2 pathways in NMuMG and MDAMB231 cells. Based on these findings, our results establish novel roles for VCAM-1 in human breast tumor carcinogenesis.

## 2. Results and Discussion

### 2.1. Results

#### 2.1.1. Evaluation of *VCAM-1* Expression in Primary Human Breast Cancer

**Figure 1.**
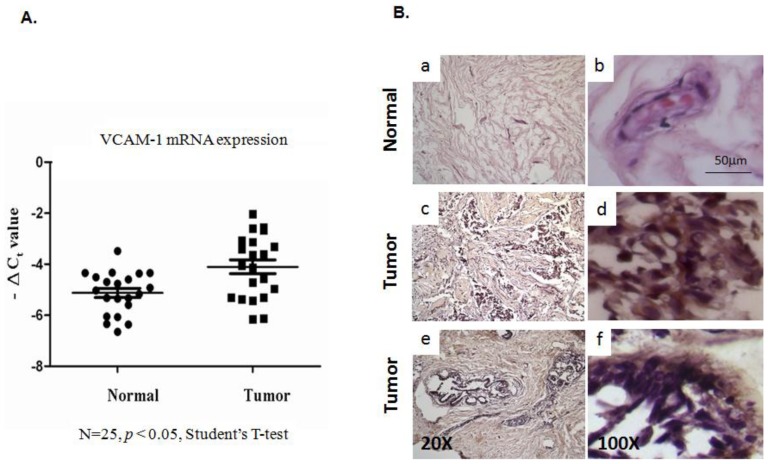
(**A**) RT-qPCR analysis of *VCAM-1* expression in breast tumor tissues (tumor stage 1 + 2 AB and 3) and noncancerous controls; (**B**) **a**,**b**, Representative IHC staining of VCAM-1 in normal breast tissues; **c**–**f**, VCAM-1 expression in human breast tumor tissues. The magnifications are indicated.

To investigate the function of *VCAM-1* in breast tumors, we assembled through collaborations samples from breast cancer cell lines and more than 25 primary breast tumor sample pairs for this analysis. We initially evaluated *VCAM-1* expression in a series of breast tumor specimens by quantitative RT-qPCR using total RNA isolation from fresh frozen breast tumor tissues. As shown in Figure 1A, our analysis of *VCAM-1* gene expression at the RNA level using RT-qPCR suggested that *VCAM-1* could be the critical activated gene during breast carcinogenesis. To examine whether increases at the RNA level translated to over-expression at the protein level and to determine the distribution of *VCAM-1*-expressing cells in primary tumor tissues, we performed immuno-histochemistry (IHC) analysis to detect VCAM-1 protein expression in primary breast tumor sections. We observed predominantly cytoplasmic staining for VCAM-1 protein in breast tumor cells (Figure 1B), consistent with the pattern observed in previous reports, showing that some tumor cells appear to express the *VCAM-1* gene during breast carcinogenesis [37]. By contrast, there was very low or no *VCAM-1* expression detectable in breast ductal epithelial cells from normal breast tissues.

#### 2.1.2. Proinflammatory Cytokine-Induced VCAM-1 Over-Expression in Normal and Malignant Breast Epithelial Cells

**Figure 2.**
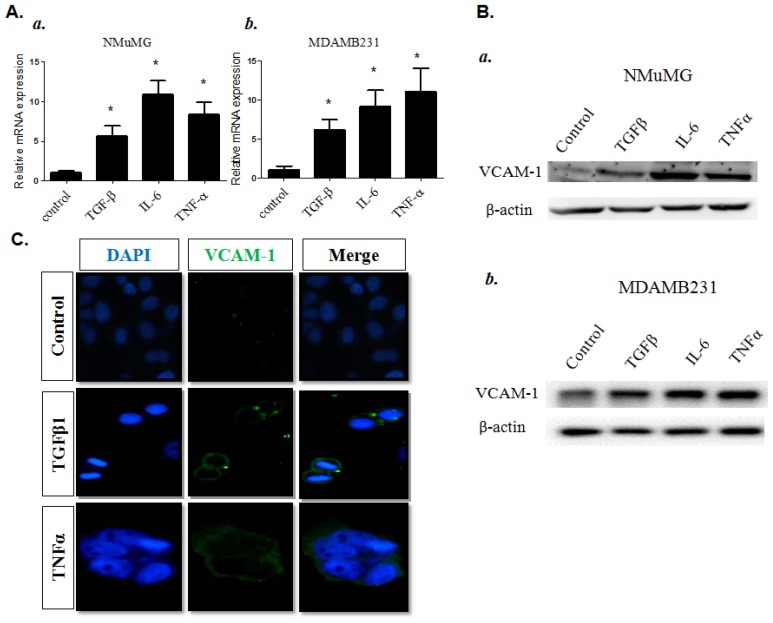
(**A**) RT-qPCR analysis for *VCAM-1* mRNA expression in **a**, NMuMG and **b**, MDAMB231 cells after treatment with control (1× PBS), TGFβ (5 ng/mL), TNFα (10 ng/mL), or IL-6 (1 ng/mL) overnight. Columns, mean of triplicate samples; bars, SE. * *p* < 0.01; (**B**) VCAM-1 protein expression in **a**, NMuMG and **b**, MDAMB231 cells by western blotting; (**C**) IF staining for *VCAM-1* expression in MDAMB231 cells with or without cytokine treatments. DNA was visualized with DAPI staining.

In general, carcinogenesis may start from an inflammatory response, which produces many different inflammatory cytokines from resident tissue cells and by infiltrating defense immune cells to regulate tumorigenesis during the different phases of tumor development, *i.e.*, initiation, promotion and progression. To study the effect of these inflammatory cytokines on *VCAM-1* expression in breast cancer cells, we analyzed the gene expression of *VCAM-1* by RT-qPCR in NMuMG, normal mouse mammary epithelial cells, and MDAMB231 breast cancer cells, under normal or inflammatory conditions for treatment with several inflammatory cytokines mimicking the *in vivo* proinflammatory tumor environment. As shown in Figure 2A, our RT-qPCR results showed that increasing *VCAM-1* expression was detected in NMuMG and MDAMB231 cells after different inflammatory cytokine treatments (Figure 2A). The relative increased levels of VCAM-1 protein expression in NMuMG and MDAMB231 cells induced by several inflammatory cytokines were further confirmed by western blot analysis (Figure 2B). Furthermore, immunofluorescence staining for VCAM-1 demonstrated that *VCAM-1* was strongly and ubiquitously detected on the cell surface and in the cytoplasmic regions after exposure of MDAMB231 cells to IL-6 or TGF-β1 cytokine (Figure 2C).

#### 2.1.3. Effect of VCAM-1 Over-Expression on NMuMG Cell Migration

**Figure 3.**
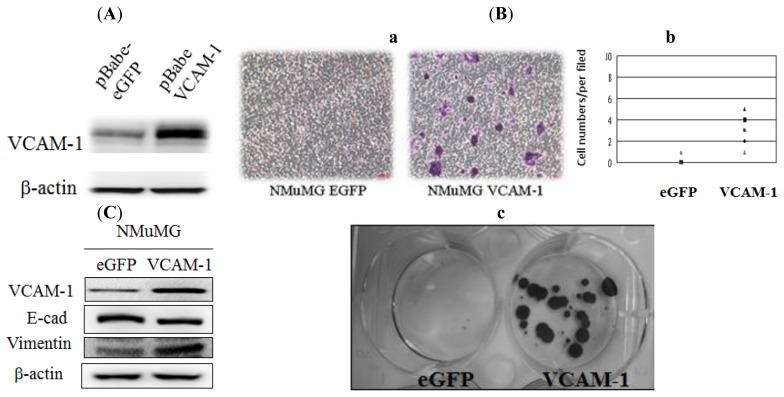
(**A**) Western blot VCAM-1 protein expression in NMuMG cells transfected with the pBabe-eGFP or the pBabe-VCAM-1 plasmid. β-actin results indicate similar sample loads; (**B**) **a**, Transwell migration analysis of NMuMG cells (control and *VCAM-1* over-expression) towards 10% FBS + insulin. Photo images are representative fields of the migration of VCAM-1 over-expressing cells and the control group after crystal violet dye staining; **b**, The cell numbers of migrated NMuMG (eGFP control) and VCAM-1 NMuMG cells from the upper insert to the bottom surface of the lower well under a light micropore (100×) after overnight incubation; **c**, Migrated NMuMG VCAM-1 cells in the lower wells showed high clonogenic ability after 2 weeks of incubation; (**C**) EMT-related protein expression in eGFP control and VCAM-1 over-expressing NMuMG cells was determined by Western blot analysis.

To evaluate the effect of *VCAM-1* up-regulation by epithelial tumor cells themselves on breast tumorgenesis, we first selected NMuMG cells isolated from the mammary glands of Namru mice to study its effects *in vitro*. We generated a pBabe retrovirus construct expressing human *VCAM-1* (see Materials and Methods). Then NMuMG cells were infected with pBabe*VCAM-1*-expressing virus or control pBabe eGFP-expressing virus and cultured in regular media. The pBabeVCAM-1 and pBabe eGFP vector-transfected stable clones were established and characterized for the expression of *VCAM-1* at both the mRNA and protein levels (Figure 3A). We first assayed NMuMG VCAM-1 cells and eGFP control cells for cell proliferation and invasion *in vitro*. Our data showed that over-expression of VCAM-1 alone in NMuMG cells did not affect cell proliferation significantly (data not shown). However, we found that over-expression of VCAM-1 in NMuMG cells increases cell adhesion and invasion in a transwell migration assay when we compared the number of cells from the underside of the transwell inserts and counted the mean number of cells migrating to the bottom wells (Figures 3B and S1b). We also observed that NMuMG VCAM-1 cells migrating to the bottom wells can continue to proliferate indefinitely and form a large colony from a single cell, unlike control cells (Figure 3B). Immunoblot analysis showed that over-expression of VCAM-1 in breast cancer cells induces an EMT-like phenotype by repressing the expression of E-cadherin and increasing the expression of the mesenchymal marker vimentin in NMuMG cells (Figure 3C).

#### 2.1.4. Down-Regulation of *VCAM-1* Expression by siRNA or shRNA Inhibits Proliferation and Migration of MDAMB231 Cells

**Figure 4.**
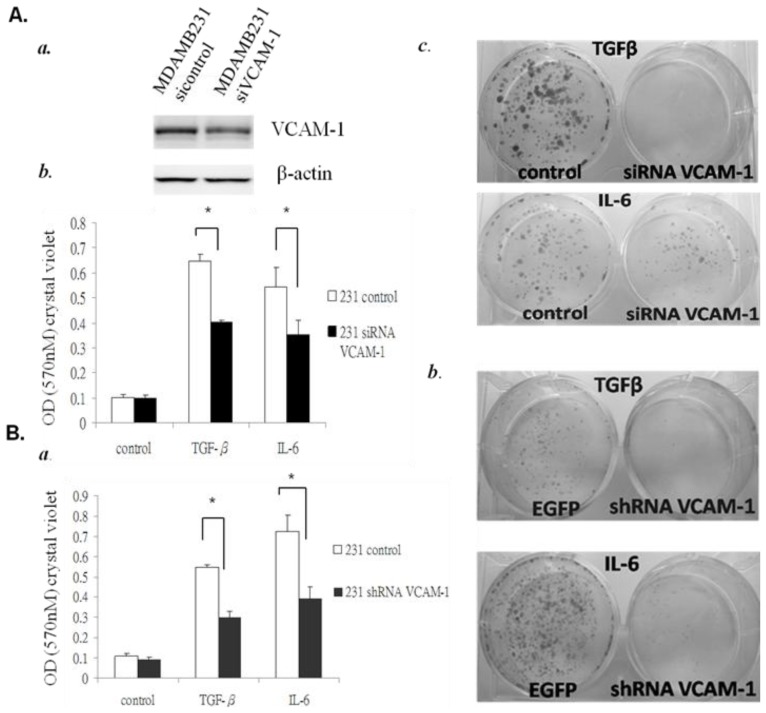
(**A**) **a**, Western blot analysis of MDAMB231 *VCAM-1* siRNA cells showed reduced VCAM-1 expression after IL-6 treatment compared with control cells; **b**, *VCAM-1* siRNA knockdown blocks TGF-β1 or IL6-induced MDAMB231 cell migration and colony formation. The lower chamber was filled with 2 mL regular medium (normal) or contained TGFβ1 (5 ng/mL) or IL-6 (1 ng/mL) as a chemoattractant; **c**, Representative photos showed the influence of VCAM-1 siRNA on the number of colony-forming MDAMB231 cells as evaluated by clonogenic assay; (**B**) The inhibition of migration and colony formation by *VCAM-1* knockdown was confirmed by shRNA knockdown stable clones. **a**,**b**, similar results confirmed the influence of *VCAM-1* shRNA knockdown on the migratory ability and number of colony-forming MDAMB231 cells. Columns, mean of triplicate samples; bars, SE; *p*-value was determined by student’s *t*-test (* *p* < 0.01).

**Figure 5.**
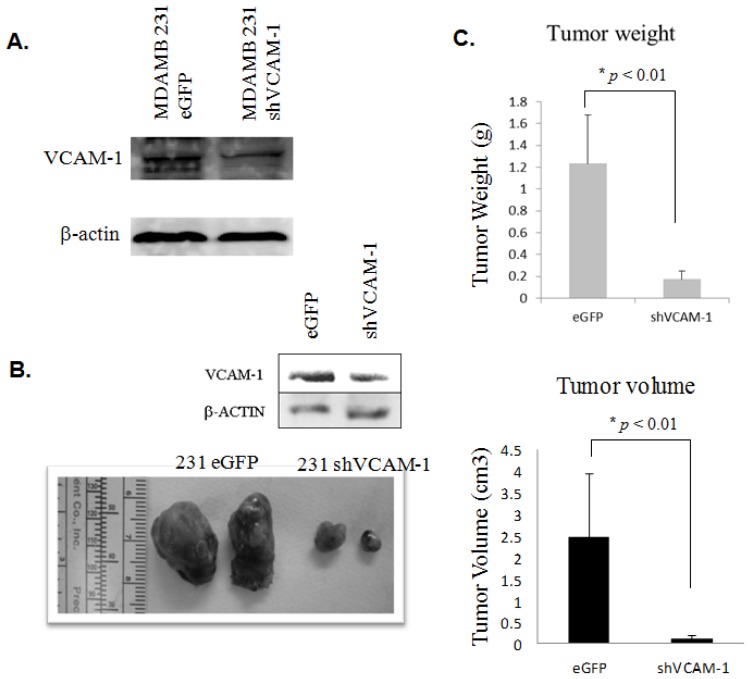
(**A**) The level of VCAM-1 protein expression in stable transfection clones was determined by western blotting; (**B**) Tumors were established using MDAMB231 *eGFP* and *VCAM-1* shRNA knockdown clones implanted by s.c. injection and analyzed after eight weeks. Western blot analysis was used to confirm *VCAM-1* knockdown efficacy in xenograft tumors (upper insert pictures); (**C**) Tumor weights were measured after autopsy and tumor volumes were calculated using the formula 4/3π*r*_1_^2^*r*_2_ (*r*_1_ < *r*_2_) as described in Materials and Methods. Mean ± SE (*n* = 6). * *p* < 0.01.

To further determine whether *VCAM-1* regulates the tumorigenesis and migration of malignant breast cancer cells, we used RNAi (for transient effects) and lentiviral shRNA construct (for long-term effects) to down-regulate *VCAM-1* expression in MDAMB231 breast cancer cells, a highly metastatic breast cancer cell line. Western blot analysis confirmed that VCAM-1-specific siRNAs effectively suppressed *VCAM-1* expression in MDAMB231 cells, whereas a control siRNA had no effect (Figure 4A). We also determined that shRNA-mediated *VCAM-1* knockdown was effective (Figure 5A). Next, we examined whether suppression of *VCAM-1* affects proliferation of breast cancer cell lines *in vitro*. We found that VCAM-1-specific siRNAs substantially reduced the proliferation of MDAMB231 breast cancer cells 2–3 fold in cell proliferation assays, whereas the control siRNAs had no effect. (Figure S1a).

To further examine the effect of VCAM-1 on breast cancer cell invasive ability, we used both *VCAM-1* siRNA and *VCAM-1* shRNA vectors to knock down *VCAM-1* expression in MDAMB231 and performed *in vitro* transwell analysis to compare the invasive abilities of *VCAM-1* knockdown and control cells. In the transwell migration assays, we added TGFβ1 (5 ng/mL) or IL-6 (1 ng/mL) to serve as a source of chemoattractant in the bottom wells to determine the involvement of VCAM-1 on TGFβ1 or IL-6 mediated regulation of tumor cell migration. After overnight incubation, our results indicated that the suppression of *VCAM-1* (either by specific *VCAM-1* siRNA or *VCAM-1* shRNA) in MDA-MB-231 cells inhibits TGFβ1 or IL-6 mediated regulation of tumor cell migration. Quantitative analysis of the number of *VCAM-1* siRNA cells migrated to the lower side of the chambers using 0.5% crystal violet staining showed a reduction of cell migration by 1.5 fold compared to cells transfected with control siRNA (Figure 4A,B). When we further continued to maintain and grow the cells that migrated to the bottom wells for two more weeks, we observed that *VCAM-1*-expressing cells showed a stronger ability to proliferate and form colonies compared to *VCAM-1* knockdown cells (Figure 4A,B).

#### 2.1.5. Knockdown of *VCAM-1* Inhibits the Growth of Human MDAMB231 Breast Cancer Xenografts in SCID Mice

Next, we wondered if our results above for the *in vitro* effects could be translated into an *in vivo* model. To confirm the effect of VCAM-1 on breast carcinogenesis *in vivo*, B6 SCID mice were subcutaneously injected in two flanks with MDAMB231 cells stably transfected with *VCAM-1* shRNA or *eGFP* shRNA control cells. Two months later, considerable gross tumor enlargement was found in mice injected with the control vector and *VCAM-1* shRNA transfected cells before mice were humanely sacrificed and autopsies were performed (Figure 5B). As expected, *VCAM-1* knockdown MDAMB231 cells showed 2–3 fold reduced tumorigenicity *in vivo* compared to control groups (Figure 5C). Meanwhile, western blot assays confirmed that *VCAM-1* shRNA effectively suppressed *VCAM-1* expression in MDAMB231 xenograft tumor samples (Figure 5B upper insert).

#### 2.1.6. *VCAM-1* Expression Enhances the Chemoresistant Phenotype in Breast Cancer

**Figure 6.**
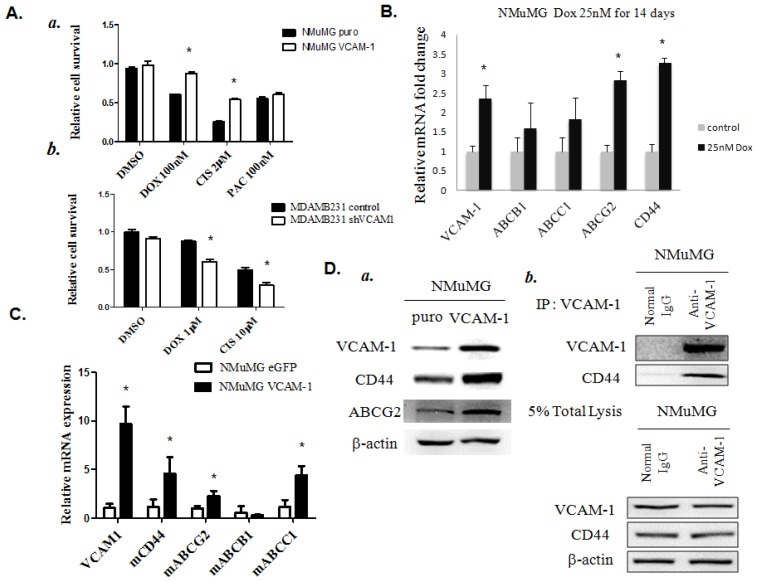
(**A**) *VCAM-1* over-expression contributes to increased chemoresistance in **a**, NMuMG and **b**, MDAMB231 cells by MTT analysis. *S*amples were analyzed after 48 h of drug treatment, * *p <* 0.01; (**B**) Increased chemoresistance markers and *VCAM-1* expression in NMuMG cells after long-term exposure to low-dose doxorubicin. Relative mRNA levels are expressed as arbitrary units normalized to the data from controls, * *p* < 0.01; (**C**) *VCAM-1* regulates migration and chemoresistance-related genes in NMuMG cells as determined by RT-qPCR, * *p* < 0.01; (**D**) Over-expression of *VCAM-1* increases *CD44* and *ABCG2* protein levels, and VCAM-1 physically interacts with *CD44* molecules in control and *VCAM-1*-over-expressing NMuMG cells as determined by western blot and immunoprecipitation analyses.

To test whether the enhanced expression of *VCAM-1* correlates with drug resistance in breast cancer cells, we investigated the effect of *VCAM-1* over-expression on the chemosensitivity of NMuMG and MDAMB231 cells by evaluating growth inhibition induced by doxorubicin (Dox), cisplatin (Cis) and paclitaxel (Pac), the standard chemo agents used in treating many types of breast cancer. The expression of *VCAM-1* resulted in increased resistance of NMuMG and MDAMB231 cells to the growth inhibiting activity of Dox and Cis (Figure 6A). Furthermore, we found increased expression of VCAM-1 following low doses and long exposure (14 days) to Dox (25 nM) in NMuMG cells, which implies that the VCAM-1 molecule may be an important determinant marker of chemoresistance in breast cancer (Figure 6B). Moreover, we also detected that the elevated expression levels of cancer stem cell-like protein, CD44 and breast cancer resistance protein (BCRP, ABCG2) were accompanied by up-regulation of *VCAM-1* after stimulation with low-dose Dox (Figure 6B), leading us to wonder if there is a potential link between *VCAM-1* expression and increased transcription of the cancer stem cell (CSC) marker, CD44. RT-qPCR was performed to compare the mRNA expression levels of *CD44*, *ABCG2*, *ABCB1* and *ABCC1* between *VCAM-1* NMuMG cells and control cells (Figure 6C). Our results revealed that *VCAM-1* over-expression activates the transcription of *CD44*, *ABCG2* and *ABCC1* in NMuMG cells (Figure 6C). Meanwhile, luciferase activity assay and western blot analysis also confirmed that the levels of *CD44* and *ABCG2* were increased in NMuMG VCAM-1 cells compared to controls (Figures 6D and S2). Similar results were obtained by silencing *VCAM-1* in MDAMB231 cells (Figure S3). In addition, we also found that endogenous VCAM-1 could interact with CD44 in NMuMG cells by using an immunoprecipitation assay (Figure 6D).

### 2.2. Discussion

As early as 1863, Rudolf Virchow *et al*. [38] first identified leucocytes in the tumor stroma and neoplastic epithelium tissues and made a connection between inflammation and tumors. This “lymphoreticular infiltrate” may reflect the basis of cancer at sites of chronic inflammation, in part based on his hypothesis that some irritants causing inflammation may actually promote cell proliferation. During carcinogenesis, tumor inflammatory microenvironments are infiltrated by multiple proinflammatory cytokines, and tumor cells are immersed in a local proinflammatory environment. Moreover, inflammatory cytokines may cooperate to influence tumor cell phenotypes. Once an inflammatory microenvironment becomes more complex with the sustained expression of early expressed cytokines, chemokines and adhesion molecules, these complex developmental events are difficult to mimic and reproduce in cell cultures *in vitro* [39,40]. Sometimes we need to ask ourselves whether or not the phenotypes of tumor cells that we study under cell culture conditions *in vitro* can actually represent the real physiological conditions of tumor cells *in vivo*.

In this study, we addressed the biological roles of *VCAM-1* expressed by tumor cells themselves after treatment with inflammatory cytokines. As far as we know, the VCAM-1 protein is one of the cell adhesion molecules that can be activated by proinflammatory cytokines and environmental stress in order to detect their expression by normal epithelial or tumor cells. Interestingly, our previous study applying genetically engineered mouse models of ovarian tumors and isolated mouse tumor cells and human ovarian cancer lines for proteomic analysis successfully identified potential biomarkers for ovarian cancer that could be applicable for developing early diagnostic methods [41,42]. One of the biomarkers we identified is VCAM-1, one of the 58 proteins found up-regulated in the mouse plasma and enriched in ovarian cancer cells, which mediates a multitude of processes including endothelial cell activation, ECM remodeling, cell migration/invasion, adhesion, inflammation and immune surveillance during carcinogenesis [43]. Next, when we employed our Kras/Pten ovarian cancer animal models to isolate primary tumor cells from *ascites* fluid; our survey of the side population of mouse ovarian cancer provided the first clues identifying the *VCAM-1* gene as an important target for regulating/controlling cancer stem-like cell activity in ovarian cancer (unpublished work [44]). Furthermore, the Oncomine database, a cancer microarray database containing datasets derived from many microarray studies, provides evidence that VCAM-1 is significantly up-regulated (*p* ≤ 10^−4^) in various cancer types (tumor *vs.* normal), including brain, breast, ovarian and esophageal carcinomas [45]. Together, all these interesting findings imply that VCAM-1 may be critical for maintaining tumor homeostasis, driving tumor progression, and mediating tumor metastasis. These findings encouraged us to pursue a better understanding of the functions of VCAM-1 in human carcinogenesis.

A recent study reported that treatment of LP9 mesothelial cells with *VCAM-1* siRNA affects ovarian cancer cell mesothelial invasion and metastatic progression. Treatment of tumor-bearing mice with function-blocking anti-*VCAM-1* antibodies increases median survival by 30% and decreases tumor burden [42]. In support of our finding, a recent report indicated that breast cancer cells with high expression of *VCAM-1* control osteoclast cells and promote bone metastasis [46]. However, little is known about the effects of *VCAM-1* on tumor cells themselves. Thus, in the present study we elected to use breast cancer cell lines as the model cell lines to dissect the functional roles of *VCAM-1* activation in breast tumor. We first confirmed the up-regulation of *VCAM-1* expression by RT-qPCR analysis in human breast cell lines and in normal and malignant breast tissues. In tissues, malignant proliferative lesions of the breast showed increased levels of VCAM-1 which were higher than those in normal breast tissue by IHC analysis. Subsequently, our study further provided evidence showing the increased expression of *VCAM-1* in many breast cancer cell lines after stimulation by inflammatory cytokines and environmental stress. These results are consistent with a previous observation that the cell adhesion molecule VCAM-1 is induced by TNF-α and may favor adhesive interactions with leukocytes, thus facilitating leukocyte tumor infiltration in breast carcinoma [47]. Meanwhile, bioinformatic analysis of the 5′ upstream promoter of VCAM-1 provided strong evidence of a number of putative binding sites for inflammatory cytokines or environmental stress induced transcriptional regulators, including SP1, STAT, NFκB, SMAD, P53 and HIF1α different transcriptional factors binding sites (Figure S4).

We next used an NMuMG cell line derived from normal mouse mammary epithelial cells to over-express *VCAM-1* and examine the biological functions of *VCAM-1* involved in mammary epithelial cell proliferation and migration. The over-expression of *VCAM-1* in NMuMG cells increased cell adhesion and migration. However, *VCAM-1* over-expression alone did not result in the increased proliferation of NMuMG cells. Furthermore, our studies showed that knockdown of *VCAM-1* expression by siRNA in MDAMB231 cells inhibited MDAMB231 cell growth *in vitro* and *in vivo*, and suppressed TGF-β1 or IL-6-stimulated MDAMB231 cell migration *in vitro*. The detailed mechanism by which knockdown of *VCAM-1* can significantly attenuate breast tumor cell migration may be due to suppression of IL-6 or TGF-β1 induced EMT. Our transcriptional analyses revealed various EMT related genes such as E-cadherin and vimentin which seem to be associated with *VCAM-1* status, suggesting the critical role of *VCAM-1* in regulating the EMT program during breast tumor progression. Meanwhile, the lack of a proliferation-promotion effect in the *VCAM-1* over-expressing NMuMG cells may suggest that the enhanced proliferation of VCAM-1 signaling may need to collaborate with other oncogenic pathways in breast cancer cells. However, the detailed mechanism of such interactions still requires further investigation.

Several studies have indicated that long-term exposure to low concentrations of cytotoxic drugs can lead to the development of a stable chemoresistant phenotype of tumor cells. Moreover, a new study has proposed that cell populations with cancer stem cell (CSC) characteristics increases after prolonged continuous selection for doxorubicin resistance in breast cancer [48]. In the present study, we showed that *VCAM-1* expression can be increased in NMuMG cells after exposure to low-dose doxorubicin for two weeks. This result strongly suggests that VCAM-1 may confer chemoresistance to anticancer drugs such as doxorubicin or cisplatin. Additionally, this study is the first to report a critical role for *VCAM-1* in regulating the CD44 CSC molecule and multiple drug resistant gene, *ABCG2*. Moreover, we also provided the first empirical evidence that *VCAM-1* and *CD44* are associated through direct interaction by using immunoprecipitation analysis. The cell surface marker CD44 has been implicated in a number of important biological processes, including lymphocyte homing, tumor metastasis, and chemoresistance [49,50]. VCAM-1 seems to function as a transcriptional activator for *CD44*, and our data implies that *VCAM-1-CD44* interaction could enhance the activation of PI3K/Akt downstream signaling pathways to increase the chemoresistance of breast cancer cells. Further studies will be required to validate the association between *VCAM-1* activation and the expression of *CD44* in clinical specimens from patients with various stages of breast cancer.

## 3. Experimental Section

### 3.1. Cell Culture, Tumor Tissues, Chemo Drugs Treatment, RNA Isolation and cDNA Synthesis

Mouse normal mammary epithelial cells were purchased from ATCC and the MDA-MB-231, and HEK293T cell lines were provided by Dr. Sam Thiagalingam at Boston Medical Center (Boston, MA, USA) and grown as described previously [51]. Twenty-five pairs of breast tumors and adjacent noncancerous tissues were available for the study. For each case, tumor samples with matched adjacent benign tissue were collected during surgical resections at the Kaohsiung Chung-Ho Hospital Clinic (Kaohsiung, Taiwan) between 2009 and 2010. Surgically resected tumor samples were immediately snap-frozen and shipped within 24 h in dry ice, and subsequently stored in liquid nitrogen. (For detailed information on the clinicopathological characteristics of patients see Table S1). The study was approved by the Institutional Review Board of Kaohsiung Medical University (Kaohsiung, Taiwan). Sections from each specimen were examined by pathologists and graded histologically. Cytokine treatments, RNA isolation and cDNA synthesis from cell lines and tumor samples were carried out using previously described procedures [52,53].

### 3.2. Plasmid Construction

To generate pBabe-puro-*VCAM-1* plasmid, the *VCAM-1* cDNA was excised from pOTB7 plasmid (pOTB7-*VCAM-1* was purchased from Open Biosystems Mammalian Gene Collection (MGC), Huntsville, AL, USA) using I-CeuI/XhoI digestion followed by Klenow enzyme reaction, and then ligated into BamHI/XhoI-digested pBabe-puro vector. All plasmids were verified by DNA sequencing (service provided by Genomics Co., Taipei, Taiwan).

### 3.3. Retroviral Production and Infection of Target Cells

Retrovirus was generated by cotransfection of pBabe-eGFP empty vector or pBabe-puro-VCAM-1 along with pVSV-G (envelope) and pVSV-GP (packaging) plasmids in 293T cells. Target cells were infected overnight with 4 mL of virus-containing medium in the presence of 10 μg/mL polybrene. The next day, cells were cultured in fresh medium and allowed to grow for another 24 h. After replacement of fresh medium, cells were selected with 2 μg/mL puromycin for 14 days, and positive clones were isolated and used for further assays.

### 3.4. Oligonucleotide Transfection

The *VCAM-1* siRNA (*VCAM-1* siRNA (h): sc-29519) and control siRNA (sc-37007) were purchased from Santa Cruz Biotech (Santa Cruz, CA, USA). SiRNA are targeted duplexes that inhibit the endogenous *VCAM-1* expression. Cells were transfected with 200 nM of the indicated oligonucleotide using Lipofectamine 2000 reagent (Invitrogen, Carlsbad, CA, USA). Forty-eight hours after transfection, cells were plated for migration and invasion assays after the indicated treatments.

### 3.5. Lentivirus Production and Shrna for Gene Knockdown

All the plasmids required for shRNA lentivirus production were purchased from the National RNAi Core Facility, Academia Sinica, Taipei, Taiwan. The pLKO.1-shRNA vectors used for knockdown of *VCAM1* were TRCN0000123170 (*VCAM1*) and TRCN0000123172 (*VCAM1*). The pLKO.1-shEGFP control plasmid was TRCN0000072190 (EGFP). The Lipofectamine 2000 reagent (Invitrogen, Carlsbad, CA, USA) was used for lentiviral production in 293T cells with a packaging construct (pCMV-ΔR8.91), an envelope construct (pMD.G), and different shRNA or rescue constructs, according to the protocol on the RNAi Core website (http://rnai.genmed.sinica.edu.tw/webContent/web/protocols).

### 3.6. Western Blot and Immunofluorescence

Western blot and immunofluorescence were performed as described previously. Primary antibodies used were anti-VCAM-1 (sc-8304), anti-E-cadherin (sc-8426), anti-vimentin (sc-7557), anti-ABCG2 (sc-25156), anti-CD44 (sc-18849) (Santa Cruz Cell Signaling, Santa Cruz, CA, USA) and mouse anti-β-actin (Novus Biologicals, Littleton, CO, USA) [54,55].

### 3.7. Quantitative RT-PCR Analysis

Total RNA prepared from samples was used for cDNA synthesis. PCR amplification was done essentially as described above, and the results of the delta CT measurements were described in detail previously [54]. Primer sequences used for real-time qPCR here were as follows: mouse *VCAM-1* forward, 5′-CCGGCATATACGAGTGTGAA-3′, and reverse, 5′-GATGCGCAGTAGAGTGCAAG-3′. human *VCAM-1* forward, 5′-ATTGGGAAAAACAGAAAAGAG-3′, and reverse, 5′-GGCACATTGA CATAAAGTC-3′. Mouse *ABCB1* forward, 5′-CAACATCCACCAGTTCATCG-3′, and reverse, 5′-CTGATGTTGCTTCGTCCAGA-3′. Mouse *ABCC1* forward 5′-TTGAGGGTGGAGAAAAGGTG-3′, and reverse, 5′-GATCTTGAAGCGCAGGTTGT-3′. Mouse *ABCG2* forward 5′-ATAGCCACAGGC CAAAGTGT-3′, and reverse, 5′-GAAGCCATATCGAGGAATGC-3′. Mouse *CD44* forward 5′-TG GATCCGAATTAGCTGGAC-3′, and reverse, 5′-TACTATTGACCGCGATGCAG-3′. These experiments were independently repeated three times and each treatment consisted of triplicate samples.

### 3.8. Transient Transfections and Luciferase Reporter Assays

Transient transfections and luiferase reporter assays were performed as described previously [52,55].

### 3.9. Cell Proliferation Assay

For cell growth assays, 2 × 10^4^ cells were seeded in 24-well plates and incubated overnight, cells were incubated for one to five days before 5 mg/mL MTT (thiazolyl blue tetrazolium bromide) (AMRESCO LLC, Solon, OH, USA) was added to 25 μL in 500 μL McCoy’s 5A medium (Invitrogen, Carlsbad, CA, USA) and incubated for another 2 h for reaction. Medium was removed and cells treated with 200 μL DMSO (Sigma, St. Louis, MO, USA) before OD570 reading with a BioTek ELISA reader (Molecular Devices LLC, Sunnyvale, CA, USA).

### 3.10. Colony Formation Assay

Forty thousand cells were grown in 60 mm tissue culture dishes. After 14 days, the cells were washed with phosphate-buffered saline (PBS) and fixed with methanol and 0.1% crystal violet. The colonies were manually counted and then photographed.

### 3.11. *In Vitro* Cell Migration/Invasion Assay

For transwell migration assays, 2 × 10^4^ to 5 × 10^4^ cells were plated in the top chamber with a non-coated filter membrane (6-well insert; pore size, 8 μm; BD Biosciences, San Jose, CA, USA). Cells were plated in McCoy’s 5A medium with low serum, and the bottom medium was supplemented with 10% FBS or cytokines. The cells were incubated for 24 h and cells that did not migrate or invade through the pores were removed by a cotton swab. Cells on the lower surface of the membrane were counted under the microscope (100×) and stained with crystal violet for representative images. The crystal violet was further dissolved in 10% acetic acid and absorbance was measured at 570 nm for quantitative analysis [55].

### 3.12. Immunoprecipitation (IP)

Detection of the endogenous co-immunoprecipitated VCAM-1 was performed in NMuMG cells. Cells were cultured in 5% FBS medium, harvested, and lysed with ice-cold 1× RIPA buffer (50 mM Tris–HCl pH 7.4, 150 mM NaCl, 1% NP-40, 0.5% sodium deoxycholate, 0.1% SDS, and 5 mM EDTA) containing protease and phosphatase inhibitors. IP was performed using anti-mouse normal IgG or anti-VCAM-1 antibodies (Santa Cruz Biotechnology, Santa Cruz, CA, USA) in 500 μL of total cell lysate mixed with 30 μL protein A/G-agarose beads (Santa Cruz Biotechnology, Santa Cruz, CA, USA), followed by overnight incubation at 4 °C. The immune complexes were washed five times with 1 mL lysis buffer and analyzed by Western blotting.

### 3.13. Mice and Injection

Specific pathogen-free female C.B17/lcr-SCID mice, eight weeks old, were purchased from BioLASCO Taiwan Co., Ltd. (Taipei, Taiwan) for the *in vivo* tumorigenicity study. The animals were bred based on technology derived from Charles River Laboratories (Wilmington, MA, USA), maintained in the animal center at the Department of Medical Research, Kaohsiung Medical University Hospital and treated according to the institutional guidelines for the care and use of experimental animals. Mice were injected subcutaneously with 1 × 10^6^ cells in 0.1 mL into both the left and right flank of each mouse and mice were maintained for two to three months. The mice were monitored for tumor volume, overall health and total body weight. The size of the tumor was determined by caliper measurement of the subcutaneous tumor mass. Tumor volume was calculated according to the formula 4/3π*r*_1_^2^*r*_2_ (*r*_1_ < *r*_2_). Each experimental group contained six mice. At the end of three months, all mice were killed and the tumor volume and weight were measured.

### 3.14. Mice Surgery, Necropsy, Histopathology and Immunohistochemistry

Tissue samples were fixed in 10% buffered formalin for 18 h, followed by a wash with PBS and transfer to 70% ethanol. They were then embedded in paraffin, sectioned and stained with hematoxylin and eosin. *VCAM-1* over-expression was confirmed by immunohistochemistry using two different antibodies (rabbit anti-VCAM-1 (sc-8304) polyclonal antibody and anti-VCAM-1 mouse monoclonal antibody (E-10; sc-13160) from Santa Cruz Biotechnology (Santa Cruz, CA, USA), and IHC was performed as described in detail previously [55].

### 3.15. Statistical Analysis

Data are presented as mean ± S.E.M. Student’s *t*-test (two-tailed) was used to compare two groups unless otherwise indicated (χ^2^ test). *p* < 0.05 was considered significant.

## 4. Conclusions

In summary, our study aimed to elucidate the modes of *VCAM-1* over-expression and also obtain preliminary data to direct further investigations into the molecular basis of *VCAM-1* up-regulation in breast cancer and the VCAM-1 signaling networks involved in controlling EMT and chemoresistance in malignant breast tumors. The identification of pathways and target genes regulated by *VCAM-1* that promote breast cancer progression and chemoresistance could be the key to new therapies for combating breast cancer.

## Supplementary Information



## References

[1] Hartge P. (1997). Abortion breast cancer and epidemiology. N. Engl. J. Med..

[2] Mettlin C.J., Menck H.R., Winchester D.P., Murphy G.P. (1997). A comparison of breast colorectal lung and prostate cancers reported to the National Cancer Data Base and the Surveillance Epidemiology and End Results Program. Cancer.

[3] Bostner J., Ahnstrom-Waltersson M., Fornander T., Skoog L., Nordenskjold B., Stal O. (2007). Amplification of CCND1 and PAK1 as predictors of recurrence and tamoxifen resistance in postmenopausal breast cancer. Oncogene.

[4] Hui R., Campbell D.H., Lee C.S., McCaul K., Horsfall D.J., Musgrove E.A., Daly R.J., Seshadri R., Sutherland R.L. (1997). EMS1 amplification can occur independently of CCND1 or INT-2 amplification at 11q13 and may identify different phenotypes in primary breast cancer. Oncogene.

[5] Mukherjee S., Conrad S.E. (2005). c-Myc suppresses p21WAF1/CIP1 expression during estrogen signaling and antiestrogen resistance in human breast cancer cells. J. Biol. Chem..

[6] Fujita T., Liu W., Doihara H., Wan Y. (2009). An *in vivo* study of Cdh1/APC in breast cancer formation. Int. J. Cancer.

[7] Lei H., Sjoberg-Margolin S., Salahshor S., Werelius B., Jandakova E., Hemminki K., Lindblom A., Vorechovsky I. (2002). CDH1 mutations are present in both ductal and lobular breast cancer but promoter allelic variants show no detectable breast cancer risk. Int. J. Cancer.

[8] Lopez-Novoa J.M., Nieto M.A. (2009). Inflammation and EMT: An alliance towards organ fibrosis and cancer progression. EMBO Mol. Med..

[9] Bhowmick N.A., Neilson E.G., Moses H.L. (2004). Stromal fibroblasts in cancer initiation and progression. Nature.

[10] Inokuchi S., Aoyama T., Miura K., Osterreicher C.H., Kodama Y., Miyai K., Akira S., Brenner D.A., Seki E. (2009). Disruption of TAK1 in hepatocytes causes hepatic injury inflammation fibrosis and carcinogenesis. Proc. Natl. Acad. Sci. USA.

[11] Mariani F., Sena P., Marzona L., Riccio M., Fano R., Manni P., Gregorio C.D., Pezzi A., Leon M.P., Monni S. (2009). Cyclooxygenase-2 and hypoxia-inducible factor-1α protein expression is related to inflammation and up-regulated since the early steps of colorectal carcinogenesis. Cancer Lett..

[12] Popivanova B.K., Kitamura K., Wu Y., Kondo T., Kagaya T., Kaneko S., Oshima M., Fujii C., Mukaida N. (2008). Blocking TNF-α in mice reduces colorectal carcinogenesis associated with chronic colitis. J. Clin. Investig..

[13] Trapani J.A. (2005). The dual adverse effects of TGF-β secretion on tumor progression. Cancer Cell.

[14] Derynck R., Akhurst R.J., Balmain A. (2001). TGF-β signaling in tumor suppression and cancer progression. Nat. Genet..

[15] Bogiatzi S.I., Fernandez I., Bichet J.C., Marloie-Provost M.A., Volpe E., Sastre X., Soumelis V. (2007). Cutting Edge: Proinflammatory and Th2 cytokines synergize to induce thymic stromal lymphopoietin production by human skin keratinocytes. J. Immunol..

[16] Romieu-Mourez R., Francois M., Boivin M.N., Bouchentouf M., Spaner D.E., Galipeau J. (2009). Cytokine modulation of TLR expression and activation in mesenchymal stromal cells leads to a proinflammatory phenotype. J. Immunol..

[17] Yamaji-Kegan K., Su Q., Angelini D.J., Champion H.C., Johns R.A. (2006). Hypoxia-induced mitogenic factor has proangiogenic and proinflammatory effects in the lung via VEGF and VEGF receptor-2. Am. J. Physiol. Lung Cell. Mol. Physiol..

[18] Lee C.G., Link H., Baluk P., Homer R.J., Chapoval S., Bhandari V., Kang M.J., Cohn L., Kim Y.K., McDonald D.M. (2004). Vascular endothelial growth factor (VEGF) induces remodeling and enhances TH2-mediated sensitization and inflammation in the lung. Nat. Med..

[19] Hagos G.K., Carroll R.E., Kouznetsova T., Li Q., Toader V., Fernandez P.A., Swanson S.M., Thatcher G.R. (2007). Colon cancer chemoprevention by a novel NO chimera that shows anti-inflammatory and antiproliferative activity *in vitro* and *in vivo*. Mol. Cancer Ther..

[20] Surh Y.J., Na H.K. (2008). NF-kappaB and Nrf2 as prime molecular targets for chemoprevention and cytoprotection with anti-inflammatory and antioxidant phytochemicals. Genes Nutr..

[21] Polte T., Newman W., Raghunathan G., Gopal T.V. (1991). Structural and functional studies of full-length vascular cell adhesion molecule-1: Internal duplication and homology to several adhesion proteins. DNA Cell Biol..

[22] Williams A.J., Atkins R.C., Fries J.W., Gimbrone M.A., Cybulsky M.I., Collins T. (1992). Nucleotide sequence of rat *vascular cell adhesion molecule-1* cDNA. Biochim. Biophys. Acta.

[23] Woodside D.G., Kram R.M., Mitchell J.S., Belsom T., Billard M.J., McIntyre B.W., Vanderslice P. (2006). Contrasting roles for domain 4 of VCAM-1 in the regulation of cell adhesion and soluble VCAM-1 binding to integrin α4β1. J. Immunol..

[24] Feuerbach D., Feyen J.H. (1997). Expression of the cell-adhesion molecule VCAM-1 by stromal cells is necessary for osteoclastogenesis. FEBS Lett..

[25] Stanley A.C., Dalton J.E., Rossotti S.H., MacDonald K.P., Zhou Y., Rivera F., Schroder W.A., Maroof A., Hill G.R., Kaye P.M. (2008). VCAM-1 and VLA-4 modulate dendritic cell IL-12p40 production in experimental visceral leishmaniasis. PLoS Pathog..

[26] Yakubenko V.P., Lobb R.R., Plow E.F., Ugarova T.P. (2000). Differential induction of gelatinase B (MMP-9) and gelatinase A (MMP-2) in T lymphocytes upon α4β1-mediated adhesion to VCAM-1 and the CS-1 peptide of fibronectin. Exp. Cell Res..

[27] Van Dinther-Janssen A.C., Horst E., Koopman G., Newmann W., Scheper R.J., Meijer C.J., Pals S.T. (1991). The VLA-4/VCAM-1 pathway is involved in lymphocyte adhesion to endothelium in rheumatoid synovium. J. Immunol..

[28] Devine L., Lightman S.L., Greenwood J. (1996). Role of LFA-1 ICAM-1 VLA-4 and VCAM-1 in lymphocyte migration across retinal pigment epithelial monolayers *in vitro*. Immunology.

[29] Panettieri R.A., Lazaar A.L., Pure E., Albelda S.M. (1995). Activation of cAMP-dependent pathways in human airway smooth muscle cells inhibits TNF-α-induced ICAM-1 and VCAM-1 expression and T lymphocyte adhesion. J. Immunol..

[30] Gamble J.R., Bradley S., Noack L., Vadas M.A. (1995). TGF-β and endothelial cells inhibit VCAM-1 expression on human vascular smooth muscle cells. Arterioscler. Thromb. Vasc. Biol..

[31] Fukushi J., Ono M., Morikawa W., Iwamoto Y., Kuwano M. (2000). The activity of soluble VCAM-1 in angiogenesis stimulated by IL-4 and IL-13. J. Immunol..

[32] Griffioen A.W., Damen C.A., Blijham G.H., Groenewegen G. (1996). Tumor angiogenesis is accompanied by a decreased inflammatory response of tumor-associated endothelium. Blood.

[33] Lee Y.W., Kuhn H., Hennig B., Neish A.S., Toborek M. (2001). IL-4-induced oxidative stress upregulates *VCAM-1* gene expression in human endothelial cells. J. Mol. Cell. Cardiol..

[34] Jeong H.J., Lee H.H., Kim Y.S., Kim S.I., Moon J.I., Park K. (1998). Expression of ICAM-1 and VCAM-1 in renal allograft rejection. Transplant. Proc..

[35] Hemmerlein B., Scherbening J., Kugler A., Radzun H.J. (2000). Expression of VCAM-1 ICAM-1 E- and P-selectin and tumour-associated macrophages in renal cell carcinoma. Histopathology.

[36] Vasselli J.R., Shih J.H., Iyengar S.R., Maranchie J., Riss J., Worrell R., Torres-Cabala C., Tabios R., Mariotti A., Stearman R. (2003). Predicting survival in patients with metastatic kidney cancer by gene-expression profiling in the primary tumor. Proc. Natl. Acad. Sci. USA.

[37] Regidor P.A., Callies R., Regidor M., Schindler A.E. (1998). Expression of the cell adhesion molecules ICAM-1 and VCAM-1 in the cytosol of breast cancer tissue benign breast tissue and corresponding sera. Eur. J. Gynaecol. Oncol..

[38] Sansone P., Bromberg J. (2011). Environment inflammation and cancer. Curr. Opin. Genet. Dev..

[39] Ben-Baruch A. (2003). Host microenvironment in breast cancer development: Inflammatory cells cytokines and chemokines in breast cancer progression: Reciprocal tumor-microenvironment interactions. Breast Cancer Res..

[40] Raman D., Baugher P.J., Thu Y.M., Richmond A. (2007). Role of chemokines in tumor growth. Cancer Lett..

[41] Yurkovetsky Z., Skates S., Lomakin A., Nolen B., Pulsipher T., Modugno F., Marks J., Godwin A., Gorelik E., Jacobs I. (2011). Development of a multimarker assay for early detection of ovarian cancer. J. Clin. Oncol..

[42] Slack-Davis J.K., Atkins K.A., Harrer C., Hershey E.D., Conaway M. (2009). Vascular cell adhesion molecule-1 is a regulator of ovarian cancer peritoneal metastasis. Cancer Res..

[43] Pitteri S.J., JeBailey L., Faca V.M., Thorpe J.D., Silva M.A., Ireton R.C., Horton M.B., Wang H., Pruitt L.C., Zhang Q. (2009). Integrated proteomic analysis of human cancer cells and plasma from tumor bearing mice for ovarian cancer biomarker discovery. PLoS One.

[44] Cheng K.H., Dinulescu D.M. (2014). Molecular analysis of genes associated with the phenotype of stem cell-like side population in ovarian cancer cells. PLoS One.

[45] Rhodes D.R., Yu J., Shanker K., Deshpande N., Varambally R., Ghosh D., Barrette T., Pandey A., Chinnaiyan A.M. (2004). Oncomine: A cancer microarray database and integrated data-mining platform. Neoplasia.

[46] Lu X., Mu E., Wei Y., Riethdorf S., Yang Q., Yuan M., Yan J., Hua Y., Tiede B.J., Lu X. (2011). VCAM-1 promotes osteolytic expansion of indolent bone micrometastasis of breast cancer by engaging α4β1-positive osteoclast progenitors. Cancer Cell.

[47] O’Hanlon D.M., Fitzsimons H., Lynch J., Tormey S., Malone C., Given H.F. (2002). Soluble adhesion molecules (E-selectin ICAM-1 and VCAM-1) in breast carcinoma. Eur. J. Cancer.

[48] Smith L., Watson M.B., O’Kane S.L., Drew P.J., Lind M.J., Cawkwell L. (2006). The analysis of doxorubicin resistance in human breast cancer cells using antibody microarrays. Mol. Cancer Ther..

[49] Ponta H., Sherman L., Herrlich P.A. (2003). CD44: From adhesion molecules to signalling regulators. Nat. Rev. Mol. Cell. Biol..

[50] Abraham B.K., Fritz P., McClellan M., Hauptvogel P., Athelogou M., Brauch H. (2005). Prevalence of CD44^+^/CD24^−^/low cells in breast cancer may not be associated with clinical outcome but may favor distant metastasis. Clin. Cancer Res..

[51] Cheng K.H., Ponte J.F., Thiagalingam S. (2004). Elucidation of epigenetic inactivation of SMAD8 in cancer using targeted expressed gene display. Cancer Res..

[52] Papageorgis P., Cheng K., Ozturk S., Gong Y., Lambert A.W., Abdolmaleky H.M., Zhou J.R., Thiagalingam S. (2011). Smad4 inactivation promotes malignancy and drug resistance of colon cancer. Cancer Res..

[53] Chiu C.Y., Kuo K.K., Kuo T.L., Lee K.T., Cheng K.H. (2012). The activation of MEK/ERK signaling pathway by bone morphogenetic protein 4 to increase hepatocellular carcinoma cell proliferation and migration. Mol. Cancer Res..

[54] Bardeesy N., Aguirre A.J., Chu G.C., Cheng K.H., Lopez L.V., Hezel A.F., Feng B., Brennan C., Weissleder R., Mahmood U. (2006). Both p16(Ink4a) and the p19(Arf)-p53 pathway constrain progression of pancreatic adenocarcinoma in the mouse. Proc. Natl. Acad. Sci. USA.

[55] Su H.T., Weng C.C., Hsiao P.J., Chen L.H., Kuo T.L., Chen Y.W., Kuo K.K., Cheng K.H. (2013). Stem cell marker nestin is critical for TGF-β1-mediated tumor progression in pancreatic cancer. Mol. Cancer Res..

