# Supplementary material for: Activation of VCAM-1 and Its Associated Molecule CD44 Leads to Increased Malignant Potential of Breast Cancer Cells
Source: Int J Mol Sci. 2014 Feb 27;15(3):3560–79. doi: 10.3390/ijms15033560 (PMC3975354; doi:10.3390/ijms15033560)
Supplement: Supplementary file 1 [file ijms-15-03560-s001.pdf]

Supplementary Information

Table S1. Comparison of clinicopathological features in 25 breast cancer patients.

Characteristic	Number of patients
**Gender**	
Female	25
Male	0
**Age**	
<45	7
46–60	13
>61	5
**Stage**	
pT1	9
pT2	12
pT3 + pT4	4
**ER/PR**	
+	13
−	12
**Histology**	
IDC	22
ILC	2
IDC + ILC	1
**Lymph node mestalasis**	
+	14
−	11

Figure S1. VCAM-1 affects breast cancer cells proliferation and adhesion. (**A**) *VCAM-1* knockdown inhibits MDAMB231 cells proliferation *in vitro*. * $p < 0.01$; (**B**) Overexpression VCAM-1 enhances adhesion of NMuMG cells *in vitro*. * $p < 0.01$; (**C**) **a**,**b**, *VCAM-1* knockdown decreases MDAMB231 cells adhesive ability *in vitro*, * $p < 0.01$ and representative photos were taken under the light microscope (40×).

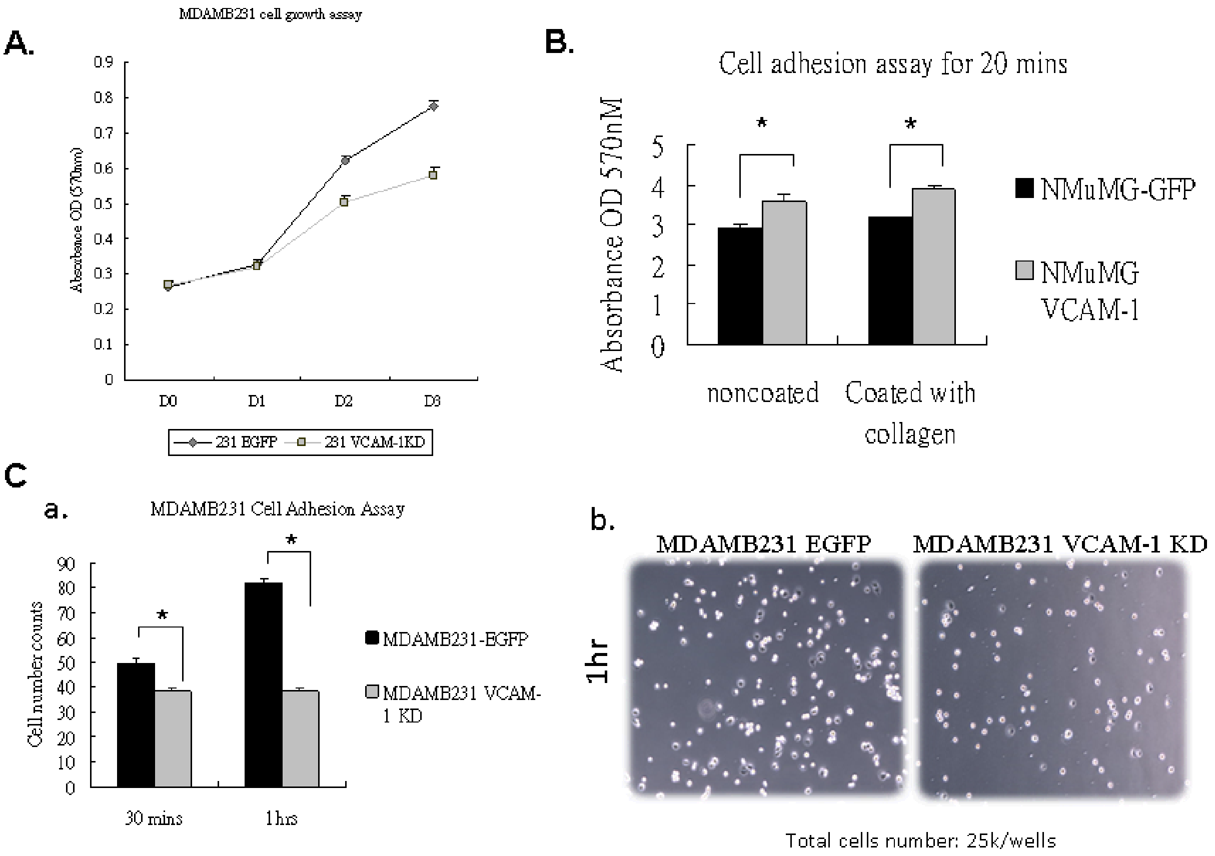

Figure S2. VCAM-1 modulates ABCG2 and CD44 promoter activity. Luciferase activity driven by the CD44 (−1262/+109) and ABCG2 promoters was compared between NMuMG VCAM-1 and NMuMG control cells. Firefly and Renilla luciferase activities were measured by the dual-luciferase reporter assay. To normalize the transfection efficiency, a fixed amount of pRL-CMV reporter, constitutively expressing the *R. reniformis* luciferase gene, was included in each sample. *Graphs* represent the mean of at least three independent experiments. Standard deviations are indicated. * $p < 0.05$.

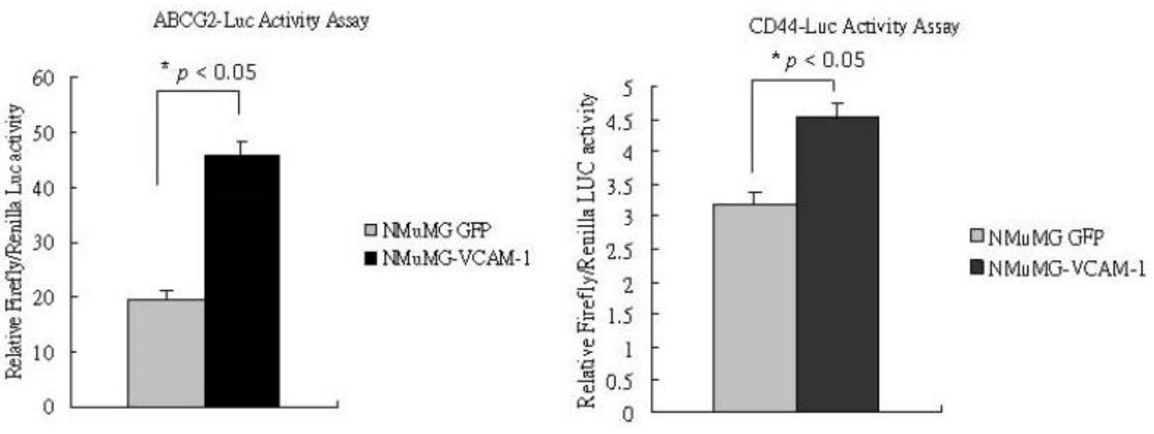

Figure S3. Knockdown of *VCAM-1* reduces CD44 and ABCG2 protein levels in MDAMB231 cells. Protein levels of VCAM-1, CD44, and ABCG2 in MDAMB231 and MDAMB231 shVCAM-1 cells were determined by Western blotting. Anti-β-actin was used as a loading control.

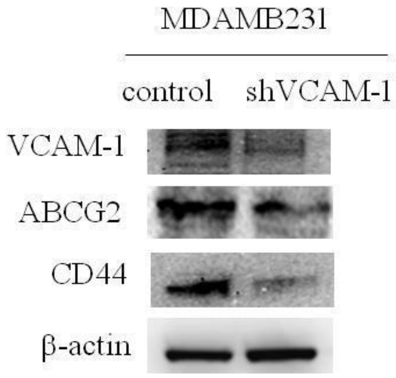

Figure S4. Schematic diagram of putative transcriptional factors binding sites in the VCAM-1 promoter. A plausible promoter region of 2.5 kb upstream to the VCAM-1 translation start site was obtained from UCSC genome database (http://genome.ucsc.edu/), and prediction of putative transcription factor binding sites was performed using MatInspector version 2.2 (http://www.genomatix.de/products/MatInspector/).

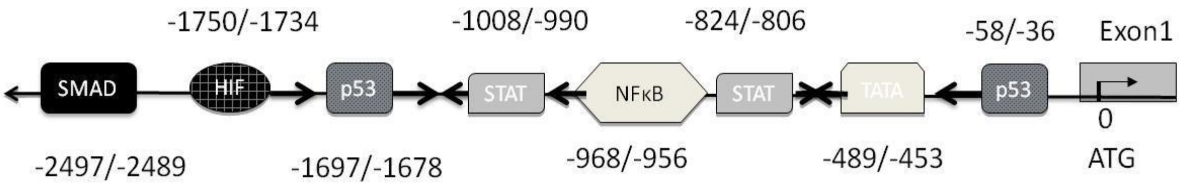

	Matrix-matched sequence
SMAD:	gGTCTtggc
TATA box:	gcttaTAAAcaagtaac
HIF:	gacctgcaCGTGtttaa
P53:	taaaCAAGtaacccagaggtcct
NFκB:	agtggctaTTCCg
STAT:	ggttTCCCcttgaagggat

© 2014 by the authors; licensee MDPI, Basel, Switzerland. This article is an open access article distributed under the terms and conditions of the Creative Commons Attribution license (http://creativecommons.org/licenses/by/3.0/).
